# The Effects of Prolonged Indoor Inhalation of Nature-Derived Odors on Menopausal Women

**DOI:** 10.3390/healthcare12161667

**Published:** 2024-08-21

**Authors:** Choyun Kim, Gayoung Lee, Chorong Song

**Affiliations:** Department of Forest Science, Kongju National University, 54 Daehak-ro, Yesan-eup, Yesan-gun 32439, Chungcheongnam-do, Republic of Korea; kcy2605@gmail.com (C.K.); 2tjduktks@naver.com (G.L.)

**Keywords:** blood pressure, middle-aged woman, nature scent, quality of sleep, stress relief, sex hormone

## Abstract

This study aimed to investigate the effects of prolonged inhalation of nature-derived odors indoors on humans. Twenty-six women participated in this study. Heart rate variability, heart rate, blood pressure, pulse rate, estradiol, testosterone, and cortisol were used as indicators of autonomic nervous system and endocrine system activities. Profile of mood state, state–trait anxiety inventory, menopause rating scale and general sleep disturbance scale were used as psychological indicators. The order was as follows: After the participants relaxed in a chair for 5 min, their heart rate variability and heart rate were measured for 3 min with their eyes closed. Subsequently, blood pressure and pulse rate were measured, salivary samples were collected for estradiol, testosterone, and cortisol analyses, and a subjective assessment was conducted. The participants received a diffuser containing fir essential oil and were instructed on its usage and precautions. Subsequently, they returned home and inhaled the fir oil for a week. After 7 days, participants revisited the laboratory for posttest measurements, conducted at the same time as the pretest. Prolonged inhalation of the fir essential oil resulted in increased estradiol concentration, decreased systolic and diastolic blood pressure, relief of menopausal symptoms, reduced anxiety levels, improved sleep quality and mood states. Prolonged inhalation of the fir essential oil induced physiological and psychological relaxation on menopausal women.

## 1. Introduction

Since modern people spend more than 80% of their time indoors [1], creating pleasant indoor environments has become an important issue. In practical terms, a comfortable indoor space not only improves work efficiency [2] and enhances quality of life [3], but also helps reduce the stress state [4,5].

Recent studies have reported that when natural elements are placed in indoor spaces, people feel comfortable and experience positive effects on their physiological and psychological relaxation [6,7,8,9]. Park et al. [6] reported that placing foliage plants indoors decreased brain activity and increased feelings of comfort. Ikei et al. [7] found that viewing roses indoors for 4 min activated the parasympathetic nervous system, which reflects a state of physiological relaxation and decreased negative emotions. Park and Mattson [8] reported that when plants were placed in hospital recovery rooms, patients perceived the environment as pleasant, and their systolic blood pressure, pain levels, anxiety, and fatigue were significantly lower than those of patients in rooms without plants. Song et al. [9] observed that elderly patients undergoing rehabilitation showed decreased sympathetic nervous system activity, reflecting reduced stress and awakening, and increased parasympathetic nervous system activity after viewing foliage plants for 1 min.

Furthermore, it has been reported in recent research that not only the presence of indoor plants, but also inhaling the scent derived from indoor plants helps human physiological and psychological relaxation. Matsubara and Kawai [10] demonstrated that the inhalation of volatile organic compounds released from cedar trees reduced the secretion of cortisol, a stress hormone. Choi et al. [11] found that when spaces were arranged for students to inhale rosemary, lemon, and peppermint scents, they experienced a positive mood and their learning performance increased. Ikei et al. [12] reported that inhaling hinoki cypress chips reduces the concentration of oxyhemoglobin in the prefrontal cortex and increases feelings of comfort. Kim et al. [13] reported that the inhalation of fir essential oil for 3 min indoors reduced sympathetic nervous system activity among university students. Hamdamian et al. [14] demonstrated a significant reduction in anxiety and pain during the first stage of labor among nulliparous women with the inhalation of Rosa damascena oil. Karimzadeh et al. [15] observed a significant decrease in pain among conscious patients admitted to the Intensive Care Unit with the use of lavender oil. Parunkul et al. [16] reported that herbal steam baths were helpful in reducing symptoms of allergic rhinitis.

Inhalation of essential oils in indoor spaces is convenient in terms of time and space, and it is also cost-effective, making it highly accessible for daily use. Additionally, smell is connected to the limbic system, which regulates emotions, and the hippocampus, which is responsible for memory [17,18]. The brain has the characteristic of remembering emotions and impressions associated with specific scents for a long time [19]; therefore, inhaling essential oils can be used as a very useful resource for relieving stress and relaxing the mind and body in daily life.

However, previous studies have examined short-term effects within 3–10 min [10,11,12,13], and research investigating long-term effects is lacking. Considering the characteristics of modern individuals who spend extensive time indoors, it is necessary to examine their long-term physiological and psychological responses to inhaling natural-derived odors. Therefore, the purpose of this study was to investigate the physiological and psychological effects of long-term inhalation of nature-derived odors.

## 2. Materials and Methods

### 2.1. Participants

This study was approved by the Institutional Review Board of the Kongju National University (IRB number: KNU_IRB_2021-67) and registered by the Clinical Research information Service (KCT0008850). The study participants were middle-aged women (45–55 years), and they were recruited through promotional posts in online communities. We conducted the study with 26 people (average age: 51.0 ± 2.9 years old, average height: 161.2 ± 4.1 cm, average weight: 59.9 ± 8.7 kg) who understood the purpose of the study and wished to participate voluntarily.

### 2.2. Experimental Design

The study had a one–group pretest–posttest design. Food and caffeine intake, which may affect the human body’s response, was prohibited 1 h before the experiment.

**Figure 1:**
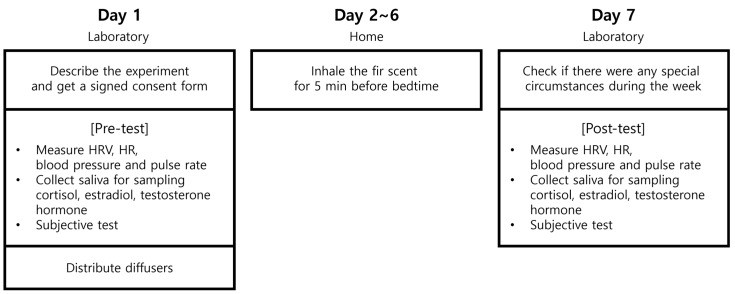
Experimental protocol.

The experimental procedure is illustrated in Figure 1. The participants arrived at the laboratory and received an explanation of the purpose and procedures of the study. The participants voluntarily signed a consent form. Pretest measurements were taken to assess the participants’ baseline status. Heart rate variability, heart rate, blood pressure, pulse rate, estradiol, testosterone, and cortisol were used as physiological measurement indicators. The order of the pretest was as follows: After the participants relaxed in a chair for 5 min, their heart rate variability and heart rate were measured for 3 min with their eyes closed. Their blood pressure and pulse rate were measured, and salivary samples were collected for estradiol, testosterone, and cortisol analyses. Following the completion of all physiological measurements, a subjective assessment was conducted.

The participants were provided with a diffuser containing fir (*Abies holophylla*) essential oil and instructed on how to use it and any precautions to be taken. They then returned home and inhaled the fir oil for a week.

After 7 days, participants revisited the laboratory for posttest measurements at the same time as the pretest. First, participants were asked questions about any specific incidents, such as illnesses or accidents, that occurred during the one-week period for monitoring purposes. After the completion of the questions, posttest measurements were conducted following the same content, procedures, and timing as the pretest.

### 2.3. Olfactory Stimulation

The fir essential oil used in this study was extracted from fir leaves using steam distillation (Kanta Enterprises Pvt. Ltd., Noida, India). The chemical composition of the essential oil used in this study is presented in Table 1.

In order for the participants to continue inhaling the fir scent at home, we made a diffuser using a container made of 4.6 × 4.6 × 7.3 (cm) square glass and five 10 cm tree branches and distributed it to participants. The participants were instructed to store the diffuser in a location that pets or infants could not reach and to place it away from direct sunlight or heat. The diffuser was placed within 2 m of the participants’ sleeping area, and the participants were requested to lie down in their sleep environment and inhale the fir essential oil for 5 min in a relaxed state before falling asleep.

### 2.4. Physiological Measurement

#### 2.4.1. Heart Rate Variability (HRV) and Heart Rate (HR)

Heart rate variability (HRV) is a method for measuring autonomic nervous system responses using variations in the intervals between heartbeats (R-R intervals) [20]. The autonomic nervous system automatically or reflexively regulates organ function, thus maintaining homeostasis in response to internal and external environmental changes [21].

In this study, R-R interval data were measured using myBeat (WHS-1, Union Tool Co., Tokyo, Japan). To analyze the frequency components of the R-R interval data, the maximum entropy method (MemCalc/win, GMS, Tokyo, Japan) was used to calculate the low-frequency (LF: less than 0.04–0.15 Hz) and high-frequency (HF: less than 0.15–0.40 Hz) components [22]. The HF component reflects the parasympathetic nervous system activity, whereas the LF/(LF + HF) ratio reflects the sympathetic nervous system activity [23].

Heart rate (HR) was measured as the number of heart beats in one min.

#### 2.4.2. Blood Pressure and Pulse Rate

Systolic blood pressure (SBP) represents the pressure exerted on the blood vessels when the heart contracts, while diastolic blood pressure (DBP) represents the pressure exerted on the blood vessels when the heart is relaxed. The pulse rate was also measured, which is the number of times the arterial wall vibrates per minute owing to contractions of the heart [24].

In this study, an oscillometric method (BPBIO330n; Inbody, Seoul, Republic of Korea) was used to measure the blood pressure. Two measurements were taken on the right upper arm of each participant. If the difference in systolic blood pressure between the first and second measurements was equal to or exceeded 10 mmHg, or the difference in diastolic blood pressure was equal to or exceeded 6 mmHg, an additional measurement was taken, and the average of the second and third measurements was used.

#### 2.4.3. Sex Hormones (Estradiol and Testosterone)

Women experience menstrual cycles due to fluctuations in sex hormone levels [25]. Estrogen and progesterone are the primary hormones that regulate the menstrual cycle. Estrogen increases during the early phase of the menstrual cycle, thickening the uterine lining, whereas progesterone increases during the later phase to maintain and develop a thickened uterine lining, preparing it for successful embryo implantation [26]. Among the estrogens, estradiol has the highest secretion and physiological activity [27]. Therefore, in this study, estradiol concentration was used as an indicator.

Testosterone is primarily associated with the development of male reproductive organs [28]. While testosterone is often referred to as a male hormone due to its higher concentration in males, it also exists in females. Testosterone promotes muscle growth, reduces fat content, and strengthens the bones [29].

#### 2.4.4. Cortisol

Cortisol is secreted by the adrenal cortex in response to stress [30]. When the concentration of serum cortisol increases, blood pressure, pulse rate, and body temperature also increase. Prolonged elevation of serum cortisol levels due to chronic stress can disrupt hormonal balance, impair immune function, induce fatigue and irregular menstrual cycles, and contribute to feelings of psychological lethargy.

Cortisol has a circadian rhythm, with higher secretion in the morning, gradually decreasing levels in the afternoon, and reaching its lowest level at night [31]. Therefore, consideration of the measurement time is crucial.

Previously, cortisol measurements were possible only using blood or urine samples. However, non-invasive saliva collection methods have recently become available [32]. When collecting saliva samples, it was necessary to restrict activities such as brushing teeth, smoking, and food intake for at least 30 min before collection.

### 2.5. Psychological Measurement

#### 2.5.1. Profile of Mood State (POMS)

The Profile of Mood State (POMS) is a questionnaire designed to evaluate various mood states [32]. It comprises 30 items, each rated on a 5-point Likert scale. This questionnaire is composed of six subscales: tension–anxiety (T–A), depression (D), anger–hostility (A–H), fatigue (F), confusion (C), and vigor (V). Each of these subscales is scored individually. Furthermore, you can calculate a total mood disturbance (TMD) score using the formula [(T–A) + (D) + (A–H) + (F) + (C) − (V)].

#### 2.5.2. State–Trait Anxiety Inventory (STAI)

The State–Trait Anxiety Inventory (STAI) is a test that measures anxiety [33]. The State Anxiety Scale consists of 20 questions assessing the current state of anxiety. Higher scores indicate higher levels of anxiety.

#### 2.5.3. Menopause Rating Scale (MRS)

The Menopause Rating Scale (MRS) is a questionnaire used to evaluate menopausal symptoms. We used a questionnaire developed by Heinemann et al. [34]. This questionnaire is composed of three subscales: psychological, somatic, and urogenital. It is rated on a 5-point Likert scale, with higher scores indicating more severe menopausal symptoms. Total scores range from 0 to 4 indicating almost no menopausal symptoms; 5 to 7, indicating mild symptoms; 8 to 15 indicating moderate symptoms, to >16, indicating severe menopausal symptoms [34].

#### 2.5.4. General Sleep Disturbance Scale (GSDS)

The General Sleep Disturbance Scale (GSDS) measures the level of sleep disturbances experienced during the past week. The Korean version developed by Choi et al. was used in this study [35]. The questionnaire consisted of 21 questions, including six subscales: difficulty initiating sleep, midsleep awakenings, poor sleep quality, too little sleep, sleepiness, and substances to aid sleep. The total score ranged from 0 to 147, with higher scores indicating higher levels of sleep disturbance.

### 2.6. Data Analysis

A total of twenty-six participants were included in the study, but six were excluded from the statistical analysis owing to errors in HRV data measurement. We used the G-power 3.1 program before starting the experiment to calculate the number of statistically valid participants. The setting values were as follows: effect size, 0.70; significance level, 0.05; power, 0.95; analysis method, means: difference from constant (one sample case). The minimum number of samples required was calculated to be 19, and this study included 20 participants, meeting the minimum required sample size.

Statistical analyses were conducted using SPSS (version 26.0; IBM Corp., Armonk, NY, USA). The significance level was set at *p* < 0.05. Paired t-tests were used for the statistical analysis of physiological measures. The Wilcoxon signed-rank test was used for statistical analysis of the psychological measures.

## 3. Results

### 3.1. Physiological Measurement

#### 3.1.1. Heart Rate Variability (HRV) and Heart Rate (HR)

No significant differences were found in the high-frequency component (pre-measurement: 189.76 ± 58.38 ms^2^, post-measurement: 170.73 ± 52.64 ms^2^), the low-frequency to high-frequency ratio (LF/(LF + HF) (pre: 0.47 ± 0.05, post: 0.51 ± 0.05), and heart rate (pre: 78.2 ± 2.2 bpm, post: 77.7 ± 2.3 bpm).

#### 3.1.2. Blood Pressure and Pulse Rate

**Figure 2:**
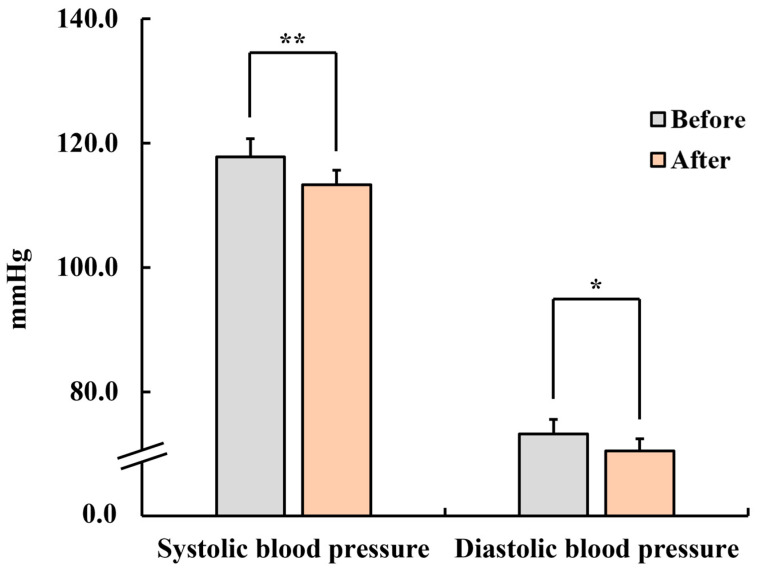
Changes in blood pressure in menopausal women after inhaling fir essential oil for a week. *n* = 26, mean ± standard error, * *p* < 0.05, ** *p* < 0.01, by paired *t*-test (one-sided).

Figure 2 shows the results of the blood pressure and pulse rate. Systolic blood pressure (pre: 117.8 ± 3.0 mmHg, post: 113.3 ± 2.3 mmHg, *p* < 0.01) and diastolic blood pressure significantly decreased (pre: 73.2 ± 2.3 mmHg, post: 70.5 ± 2.0 mmHg, *p* < 0.05). However, no significant differences were found in pulse rate between the pretest (78.0 ± 2.2 bpm) and posttest (78.6 ± 2.3 bpm).

#### 3.1.3. Sex Hormones (Estradiol and Testosterone)

**Figure 3:**
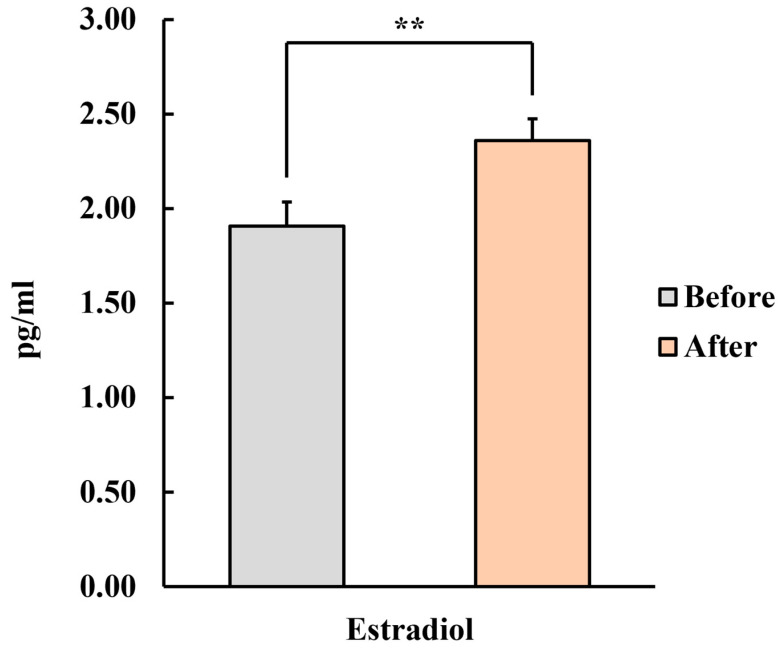
Changes in estradiol levels in menopausal women after inhaling fir essential oil for a week. *n* = 26, mean ± standard error, ** *p* < 0.01 by paired *t*-test (one-sided).

Figure 3 shows the results of the sex hormone levels. The analysis of estradiol levels showed a significant increase (pre: 1.91 ± 0.13 pg/mL, post: 2.36 ± 0.12 pg/mL, *p* < 0.01), indicating a higher concentration of estradiol after the intervention. On the other hand, no significant differences were observed in testosterone levels (pre: 96.52 ± 8.91 pg/mL, post: 98.14 ± 8.11 pg/mL).

#### 3.1.4. Cortisol

The cortisol levels were measured (pre: 1.75 ± 0.20 ng/mL, post: 1.98 ± 0.16 ng/mL), but no significant differences were found.

### 3.2. Psychological Measurement

#### 3.2.1. Profile of Mood State (POMS)

**Figure 4:**
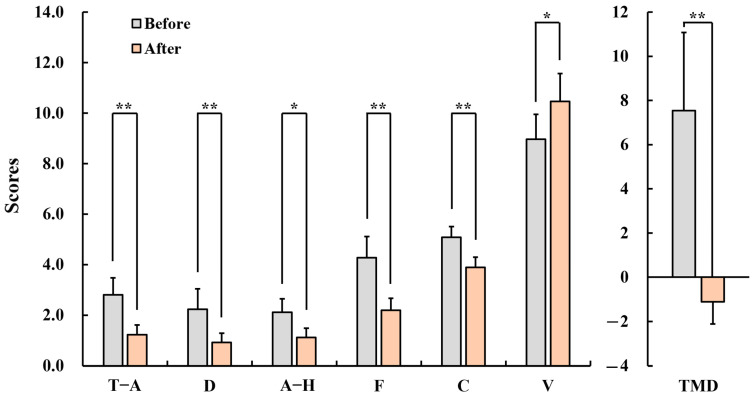
Changes in the scores of the Profile of Mood State (POMS) in menopausal women after inhaling fir essential oil for a week. T–A: tension–anxiety; D: depression; A–H: anger–hostility; F: fatigue; C: confusion; V: vigor; TMD: total mood disturbance. *n* = 26, mean ± standard error, * *p* < 0.05, ** *p* < 0.01 by Wilcoxon signed-rank test (one-sided).

Figure 4 shows the results of the POMS. The subscale scores of mood states for pre- and posttest are as follows: In the subscales of tension–anxiety (pre: 2.8 ± 0.7, post: 1.2 ± 0.4, *p* < 0.01), depression (pre: 2.2 ± 0.8, post: 0.9 ± 0.4, *p* < 0.01), anger–hostility (pre: 2.1 ± 0.5, post: 1.1 ± 0.4, *p* < 0.05), fatigue (pre: 4.3 ± 0.8, post: 2.2 ± 0.5, *p* < 0.01), confusion (pre: 5.1 ± 0.4, post: 3.9 ± 0.4, *p* < 0.01), and total mood disturbance (pre: 7.5 ± 3.5, post: 1.1 ± 2.2, *p* < 0.01) the score significantly decreased. Additionally, a significant increase in vigor was observed (pre: 9.0 ± 1.0, post: 10.5 ± 1.1, *p* < 0.05).

#### 3.2.2. State–Trait Anxiety Inventory (STAI)

**Figure 5:**
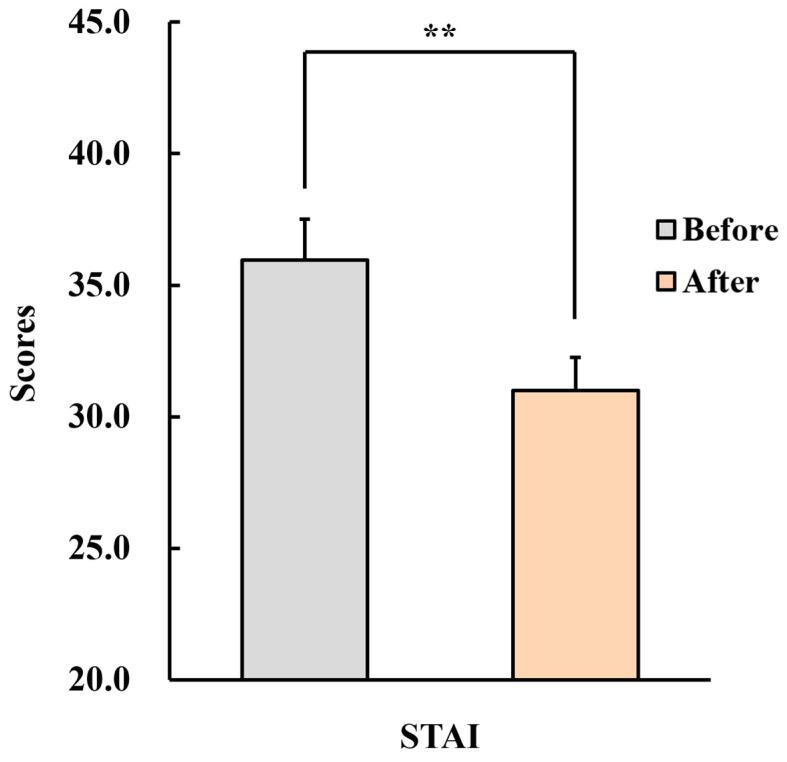
Changes in the scores of the State–Trait Anxiety Inventory (STAI) in menopausal women after inhaling fir essential oil for a week. *n* = 26, mean ± standard error, ** *p* < 0.01 by Wilcoxon signed-rank test (one-sided).

Figure 5 shows the STAI results. The score significantly decreased (pre: 36.0 ± 1.6, post: 31.0 ± 1.2, *p* < 0.01).

#### 3.2.3. Menopause Rating Scale (MRS)

**Figure 6:**
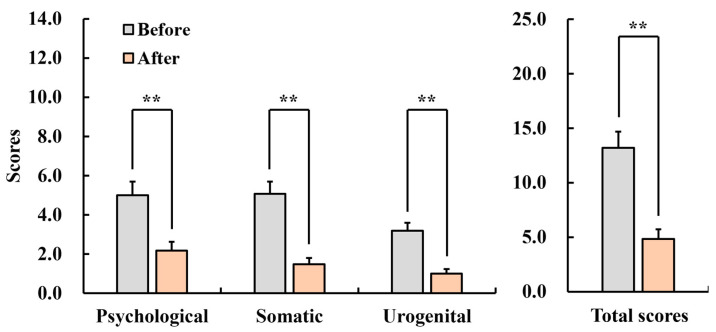
Changes in the scores of the Menopause Rating Scale (MRS) in menopausal women after inhaling fir essential oil for a week. *n* = 26, mean ± standard error, ** *p* < 0.01 by Wilcoxon signed-rank test (one-sided).

Figure 6 shows the results of the MRS. The subscale scores of menopause for pre- and posttest are as follows: In the subscales of psychological rating (pre: 5.0 ± 0.7, post: 2.2 ± 0.4, *p* < 0.01), somatic rating (pre: 5.1 ± 0.6, post: 1.5 ± 0.3, *p* < 0.01), urogenital rating (pre: 3.2 ± 0.4, post: 1.0 ± 0.2, *p* < 0.01), and total rating (pre: 13.2 ± 1.5, post: 4.8 ± 0.9, *p* < 0.01), the scores of the posttest were significantly decreased compared with scores of the pretest; therefore, menopausal symptoms were alleviated.

#### 3.2.4. General Sleep Disturbance Scale (GSDS)

**Figure 7:**
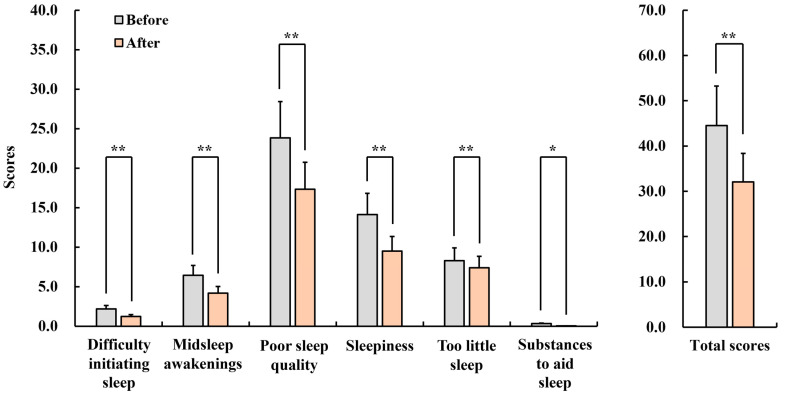
Changes in the scores of the General Sleep Disturbance Scale (GSDS) in menopausal women after inhaling fir essential oil for a week. *n* = 26, mean ± standard error, * *p* < 0.05, ** *p* < 0.01 by Wilcoxon signed-rank test (one-sided).

Figure 7 shows the results of the GSDS. The subscale scores of General Sleep Disturbance Scale for pretest and posttest are as follows: in the subscales of difficulty initiating sleep (pre: 2.2 ± 0.4, post: 1.2 ± 0.2, *p* < 0.01), midsleep awakenings (pre: 6.4 ± 1.2, post: 4.2 ± 0.8, *p* < 0.01), poor sleep quality (pre: 23.9 ± 4.6, post: 17.3 ± 3.4, *p* < 0.01), sleepiness (pre: 8.3 ± 1.6, post: 7.4 ± 1.4, *p* < 0.01), too little sleep (pre: 14.1 ± 2.7, post: 9.5 ± 1.9, *p* < 0.01), substances to aid sleep (pre: 0.3 ± 0.1, post: 0.0 ± 0.0, *p* < 0.05), and total (pre: 44.5 ± 5.7, post: 32.1 ± 6.3, *p* < 0.01), the scores were significantly decreased, and thus the quality of sleep was improved.

## 4. Discussion

This study aimed to investigate the physiological and psychological effects of prolonged inhalation of nature-derived odors indoors. HRV, HR, blood pressure, and pulse rate were used to measure autonomic nervous system activity, whereas cortisol and sex hormones were used to measure endocrine system changes. Psychological state was evaluated using four measurement indicators: POMS, STAI, GSDS, and MRS.

The blood pressure analysis showed that systolic and diastolic blood pressures had significantly decreased. This finding is consistent with those of a previous study [36]. They used massage oil and body cream mixed with lavender, masoram, ylang-ylang, and neroli for four weeks in middle-aged women with hypertension, resulting in a significant decrease in systolic blood pressure [36]. These results suggest that long-term inhalation of nature-derived substances has a positive impact on physiological relaxation and helps to reduce blood pressure.

The estradiol concentration analysis showed that the concentration had significantly increased. This finding is consistent with previous research [37], such as that by Fukui et al. [38], which reported a significant increase in estradiol levels when women with premenstrual syndrome (PMS) were exposed to the scent of saffron for 20 min. Studies further indicate that aromatherapy has also been effective in relieving menopausal symptoms. However, no significant relationship was observed in changes in estradiol levels [38]. Ghaffari et al. [38] conducted aromatherapy using fennel seed powder on middle-aged women aged 45–60 years. The study reported a significant reduction in menopausal symptoms but no significant differences in estradiol levels. We think that not enough research data exist to identify the correlation between aromatherapy and estradiol; therefore, it is considered premature to generalize. It is necessary to accumulate data through case studies, as few studies have identified its effectiveness in humans.

Subjective evaluation revealed that the long-term inhalation of fir oil improved mood and sleep quality, and reduced anxiety and menopausal symptoms. These results were consistent with those of previous studies [39,40,41]. Watanabe et al. [39] reported that when bergamot oil was inhaled for 15 min, negative mood decreased and positive mood increased. Hwang and Shin [40] reported that aromatherapy improved sleep quality and promoted sleep. Andini et al. [41] demonstrated a significant decrease in the MRS score in menopausal women who inhaled fennel essential oil twice a day (in the morning and before bed) for 5 days. Furthermore, it was confirmed that the inhalation of nature-derived oils had positive effects on psychological relaxation in middle-aged women.

Middle-aged women experience physiological changes due to a decrease in female hormones. They can experience symptoms such as hot flashes, decreased skin elasticity, and an increased risk of osteoporosis [42,43]. Also, during this period, middle-aged women may experience loneliness, anxiety, and increased depression. This can lead to insomnia and nervous system disorders [44], making the patients more susceptible to physical and mental stress. In addition, women tend to spend more time indoors than men do. According to the “Employment Characteristics of Families” report released by the Bureau of Labor Statistics (BLS) in 2020, the percentage of full-time homemakers in the United States was 22.7%, with women accounting for 96.8% and men accounting for 3.2% [45]. Considering that middle-aged women spend a lot of time indoors, the use of nature-derived scents, such as fir essential oil, will help alleviate menopausal symptoms and improve their quality of life.

This study is significant as it comprehensively verified the prolonged effects of inhaling a nature-derived odor by using various physiological and psychological indicators. The study by Kim et al. [46], which reported the short-term inhalation effect of fir, and our study share similarities in terms of the study participants being middle-aged and the stimulus being fir. The only difference was the duration of the inhalation period. Therefore, it is possible to compare the results. Short-term inhalation of fir essential oil increases parasympathetic nervous system activity; however, long-term inhalation increases female hormone levels and decreases blood pressure. We believe that the physiological effects vary depending on the period of inhalation of nature-derived odors. However, it is difficult to generalize the research findings due to the limited data; therefore, the effect of inhalation of nature-derived odors should be identified through sufficient data in the future. This study is considered an important foundational resource for interpreting human responses to the long-term inhalation of nature-derived odors.

Based on the results of this study, we propose the following directions for future research: First, because this study focused on menopausal women, future research should target diverse age groups to accumulate data and validate the results. Additionally, it is recommended that future studies should involve a larger sample size to enhance the reliability and generalizability of the findings. Second, due to difficulties in participant recruitment caused by the COVID-19 pandemic, we were unable to establish a control group that did not inhale the fir essential oil. This limitation affects this study’s internal and external validity and introduces the possibility of a placebo effect due to participants’ expectations of the fir scent. Future research should consider using a true experimental or quasi-experimental design with randomly recruited participants to enable a more rigorous comparison between experimental and control groups. Third, this study investigated the physiological and psychological relaxation of using only fir essential oil. Future research should explore the relaxation of different species of wood, such as pine, cedar, cypress, and oak, in indoor spaces. Fourth, this study was conducted over a period of one week; however, this duration may be too short to observe long-term effects. Future research should consider extending the study period to gain more comprehensive insights.

Although this study has limitations due to its experimental design, it is significant as the first comprehensive investigation into the physiological and psychological effects of the long-term indoor inhalation of nature-derived odors on menopausal women. By addressing these limitations and outlining future research directions, we believe this study will serve as an important foundational resource for subsequent research in this field.

## 5. Conclusions

This study found that the long-term inhalation of fir essential oil reduces blood pressure, increases female hormones, improves mood and sleep quality, and reduces anxiety and menopausal symptoms in menopausal women. Thus, long-term inhalation of fir essential oil has a positive impact on physiological and psychological relaxation in menopausal women.

## Figures and Tables

**Table 1:** The compounds of fir essential oil.

Compound	Detected Concentration (mg/kg)	Relative Area (%)
alpha-Pinene	226.8	22.68
Camphene	100.5	10.05
3-Carene	99.6	9.96
Limonene	88.1	8.81
p-Cymene	37.8	3.78
Camphor	36.6	3.66
gamma-Terpinene	31.6	3.16
(−)-beta-Pinene	28.4	2.84
alpha-Terpinene	13.8	1.38
alpha-Terpinolene	10.9	1.09
alpha-Terpineol	10.8	1.08
beta-Myrcene	10.3	1.03
Eucalyptol	9.5	0.95
(−)-trans-Caryophyllene	2.3	0.23
(−)-Bornyl acetate	1.0	0.10
Sum (%)		69.62

## Data Availability

The data presented in this study are available on request from the corresponding author.

## References

[1] Khajehzadeh I., Vale B. (2017). How New Zealanders distribute their daily time between home indoors, home outdoors and out of home. Kotuitui.

[2] Seppänen O., Fisk W.J., Lei Q.H. (2006). Room Temperature and Productivity in Office Work.

[3] Colenberg S., Jylhä T., Arkesteijn M. (2021). The relationship between interior office space and employee health and well-being—A literature review. Build. Res. Inf..

[4] Chen H.C., Wang C.H., Chen K.S., Chang T.L. (2014). Analysis and construction of stress relief model for healthy indoor environments. Qual. Quant..

[5] Liu G., Zou J., Qiao M., Zhu H., Yang Y., Guan H., Hu S. (2023). Stress recovery at home: Effects of the indoor visual and auditory stimuli in buildings. Build. Environ..

[6] Park S.A., Song C., Choi J.Y., Son K.C., Miyazaki Y. (2016). Foliage plants cause physiological and psychological relaxation as evidenced by measurements of prefrontal cortex activity and profile of mood states. HortScience.

[7] Ikei H., Song C., Igarashi M., Namekawa T., Miyazaki Y. (2014). Physiological and psychological relaxing effects of visual stimulation with foliage plants in high school students. Int. J. Environ. Res. Public Health.

[8] Park S.H., Mattson R.H. (2009). Ornamental Indoor Plants in Hospital Rooms Enhanced Health Outcomes of Patients Recovering from Surgery. J. Altern. Complement. Med..

[9] Song C., Ikei H., Nara M., Takayama D., Miyazaki Y. (2018). Physiological effects of viewing bonsai in elderly patients undergoing rehabilitation. Int. J. Environ. Res. Public Health.

[10] Matsubara E., Kawai S. (2014). VOCs emitted from Japanese cedar (*Cryptomeria japonica*) interior walls induce physiological relaxation. Build. Environ..

[11] Choi N., Yamanaka T., Takemura A., Kobayashi T., Eto A., Hirano M. (2022). Impact of indoor aroma on students’ mood and learning performance. Build. Environ..

[12] Ikei H., Song C., Miyazaki Y. (2018). Physiological effects of touching the wood of hinoki cypress (*Chamaecyparis obtusa*) with the soles of the feet. Int. J. Environ. Res. Public Health.

[13] Kim C., Song C. (2022). Physiological and Psychological Relaxation Effects of Fir Essential Oil on University Students. Int. J. Environ. Res. Public Health.

[14] Hamdamian S., Nazarpour S., Simbar M., Hajian S., Mojab F., Talebi A. (2018). Effects of aromatherapy with Rosa damascena on nulliparous women’s pain and anxiety of labor during first stage of labor. J. Integr. Med..

[15] Karimzadeh Z., Azizzadeh Forouzi M., Tajadini H., Ahmadinejad M., Roy C., Dehghan M. (2021). Effects of lavender and Citrus aurantium on pain of conscious intensive care unit patients: A parallel randomized placebo-controlled trial. J. Integr. Med..

[16] Tungsukruthai P., Nootim P., Worakunphanich W., Tabtong N. (2018). Efficacy and safety of herbal steam bath in allergic rhinitis: A randomized controlled trial. J. Integr. Med..

[17] Carmichael S.T., Clugnet M.C., Price J.L. (1994). Central olfactory connections in the macaque monkey. J. Comp. Neurol..

[18] Poo C., Agarwal G., Bonacchi N., Mainen Z. (2022). Spatial maps in piriform cortex during olfactory navigation. Nature.

[19] Ehrlichman H., Halpern J.N. (1988). Affect and Memory: Effects of Pleasant and Unpleasant Odors on Retrieval of Happy and Unhappy Memories. J. Pers. Soc. Psychol..

[20] Kobayashi H., Ishibashi K., Noguchi H. (1999). Heart Rate Variability; An Index for Monitoring and Analyzing Human Autonomic Activities. Appl. Hum. Sci..

[21] Janig W., Habler H.J. (2000). Specificity in the organization of the autonomic nervous system: A basis for precise neural regulation of homeostatic and protective body functions. Prog. Brain Res..

[22] Kuo T.B.J., Lin T., Yang C.C.H., Li C., Chen C.F., Chou P. (1999). Effect of aging on gender differences in neural control of heart rate. Heart Circ Physiol..

[23] Cacioppo J.T., Berntson G.G., Binkley P.F., Quigley K.S., Uchino B.N., Fieldstone A. (1994). Autonomic_cardiac_control_II_Noninvasive. Psychophysiology.

[24] Beevers G., Lip G.Y., O’Brien E. (2001). ABC of hypertension Blood pressure measurement Part I-Sphygmomanometry: Factors common to all techniques Methods of blood pressure measurement. BMJ.

[25] Cha J., Sun X., Dey S.K. (2012). Mechanisms of implantation: Strategies for successful pregnancy. Nat. Med..

[26] Barbieri R.L. (2014). The endocrinology of the menstrual cycle. Methods Mol. Biol..

[27] Devoto L., Henríquez S., Kohen P., Strauss J.F. (2017). The significance of estradiol metabolites in human corpus luteum physiology. Steroids.

[28] Lang T.F. (2011). The Bone-Muscle Relationship in Men and Women. J. Osteoporos..

[29] Vingren J.L., Kraemer W.J., Ratamess N.A., Anderson J.M., Volek J.S., Maresh C.M. (2010). Testosterone Physiology in Resistance Exercise and Training the Up-Stream Regulatory Elements. Sports Med..

[30] Michaud K., Matheson K., Kelly O., Anisman H. (2008). Impact of stressors in a natural context on release of cortisol in healthy adult humans: A meta-analysis. Stress.

[31] Edwards S., Clow A., Evans P., Hucklebridge F. (2001). Exploration of the awakening cortisol response in relation to diurnal cortisol secretory activity. Life Sci..

[32] Poll E.M., Kreitschmann-Andermahr I., Langejuergen Y., Stanzel S., Gilsbach J.M., Gressner A., Yagmur E. (2007). Saliva collection method affects predictability of serum cortisol. Clin. Chim. Acta.

[33] Spielberg C.D. (1983). State-Trait Anxiety Inventory for Adults.

[34] Berlin Center for Epidemiology and Health Research Menopause Rating Scale. https://zeg-berlin.de/expertise/diagnostics-tools/menopause-rating-scale/development/.

[35] Choi H.J., Kim S.J., Kim B.J., Kim I.J. (2012). Korean Versions of Self-reported Sleep Questionnaires for Research and Practice on Sleep Disturbance. Korean J. Rehabil. Nurs..

[36] Ju M.S., Lee S., Bae I., Hur M.H., Seong K., Lee M.S. (2013). Effects of aroma massage on home blood pressure, ambulatory blood pressure, and sleep quality in middle-aged women with hypertension. Evid. Based Complement. Alternat. Med..

[37] Fukui H., Toyoshima K., Komaki R. (2011). Psychological and neuroendocrinological effects of odor of saffron (*Crocus sativus*). Phytomedicine.

[38] Ghaffari P., Hosseininik M., Afrasiabifar A., Sadeghi H., Hosseininik A., Tabatabaei S.M., Hosseini N. (2020). The effect of Fennel seed powder on estradiol levels, menopausal symptoms, and sexual desire in postmenopausal women. Menopause.

[39] Watanabe E., Kuchta K., Kimura M., Rauwald H.W., Kamei T., Imanishi J. (2015). Effects of Bergamot (*Citrus bergamia* (Risso) Wright & Arn.) Essential Oil Aromatherapy on Mood States, Parasympathetic Nervous System Activity, and Salivary Cortisol Levels in 41 Healthy Females. Complement. Med. Res..

[40] Hwang E., Shin S. (2015). The effects of aromatherapy on sleep improvement: A systematic literature review and meta-analysis. J. Altern. Complement. Med..

[41] Andini S., Husni E., Aini E., Kasiati K., Kaur K. (2022). Effect of Fennel Aromatherapy (*Foeniculum vulgare*) on Decreasing Menopause Symptom Levels in Menopausal Women in Tunjung Village Bangkalan Regency Indonesia. Int. J. Adv. Health Sci. Technol..

[42] Dick S., DeWitt D., Anawalt B. (2002). Postmenopausal hormone replacement therapy and major clinical outcomes: A focus on cardiovascular disease, osteoporosis, dementia, and breast cancer. Am. J. Manag. Care.

[43] Tankó L.B., Christiansen C., Cox D.A., Geiger M.J., McNabb M.A., Cummings S.R. (2005). Relationship Between Osteoporosis and Cardiovascular Disease in Postmenopausal Women. J. Bone Miner. Res..

[44] Greenblum C.A., Rowe M.A., Neff D.F., Greenblum J.S. (2013). Midlife women: Symptoms associated with menopausal transition and early postmenopause and quality of life. Menopause.

[45] Bureau of Labor Statistics, United States Department of Labor NEW RELEASE; EMPLOYMENT CHARACTERISTICS OF FAMILIES 2022. https://www.bls.gov/news.release/famee.nr0.htm.

[46] Kim C., Lee G., Song C. (2023). The Effect of Short-term Inhalation of Fir Essential Oil on Autonomic Nervous Activity in Middle-aged Women. Explore.

